# Coexistence of Intravascular Excess Fluid and Reduced Renal Blood Flow in the Acute Phase of Acute Post-Streptococcal Glomerulonephritis

**DOI:** 10.31662/jmaj.2025-0069

**Published:** 2025-08-01

**Authors:** Hisakazu Majima, Osamu Uemura, Toshihiko Hattrori, Naoya Fujita, Katsumi Ushijima, Masamichi Miyoshi, Takuji Yamada, Masaki Yamamoto, Eiji Matsukuma, Takuhito Nagai, Yoshimitsu Gotoh

**Affiliations:** 1 Department of Pediatric Nephrology, Japanese Red Cross Aichi Medical Center Nagoya Daini Hospital, Nagoya, Japan; 2 Department of Pediatrics, Ichinomiya Medical Treatment & Habilitation Center, Ichinomiya, Japan; 3 Department of Nephrology, Aichi Children’s Health and Medical Center, Obu, Japan; 4 Department of Pediatrics, Yokkaichi Municipal Hospital, Yokkaichi, Japan; 5 Department of Pediatrics, Ichinomiya Municipal Hospital, Ichinomiya, Japan; 6 Department of Pediatrics, Nagoya City University West Medical Center, Nagoya, Japan; 7 Department of Pediatric Nephrology, Seirei Hamamatsu General Hospital, Hamamatsu, Japan; 8 Department of Pediatric Nephrology, Gifu Prefectural General Medical Center, Gifu, Japan; 9 Department of Pediatrics, Tajimi Municipal Hospital, Tajimi, Japan

**Keywords:** acute post-streptococcal glomerulonephritis, renal blood flow, prerenal acute kidney injury, fractional excretion of sodium, brain natriuretic peptide

## Abstract

**Introduction:**

Acute post-streptococcal glomerulonephritis (APSGN) is traditionally classified as an intrinsic form of acute kidney injury (AKI). However, previous reports suggest that its pathophysiology may resemble prerenal AKI, particularly regarding low fractional excretion of sodium (FENa) in the acute phase. This study aimed to evaluate the paradoxical coexistence of reduced renal blood flow and fluid overload in APSGN.

**Methods:**

We retrospectively analyzed patients with APSGN (≤15 years old) hospitalized between 2010 and 2019 who exhibited ≥5% weight gain and brain natriuretic peptide (BNP) ≥100 pg/mL. The acute phase was divided into three periods: peak (3 days), early recovery (2 days), and late recovery (up to 30 days). Patients with FENa and BNP recorded in at least two periods were included.

**Results:**

Among 10 patients (median age: 7 years, interquartile range: 5-7), BNP levels peaked during the acute phase and decreased in the recovery phases. Conversely, FENa was low during the peak phase but increased during recovery, despite decreasing BNP levels.

**Conclusions:**

In APSGN, FENa remained paradoxically low during the peak phase despite fluid overload (indicated by high BNP). These findings suggest that the acute phase of APSGN involves transient renal hypoperfusion and renin-angiotensin-aldosterone system activation, leading to sodium retention and volume overload. This mechanism supports the hypothesis that APSGN exhibits characteristics of prerenal AKI in its early stage.

## Introduction

**Figure 1.**
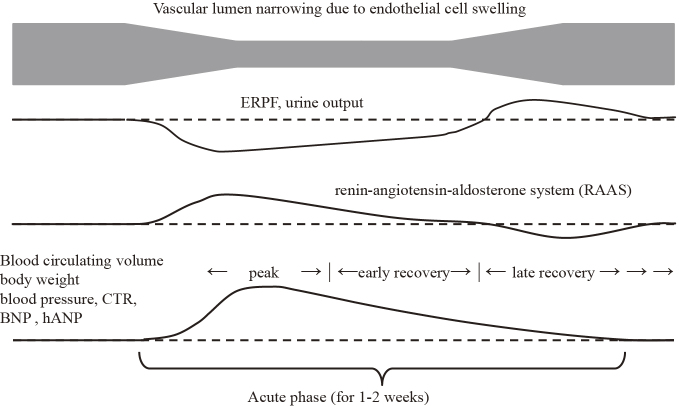
Hypothesis of time course in APSGN. APSGN: acute post-streptococcal glomerulonephritis; BNP: brain natriuretic peptide; CTR: cardiothoracic ratio; ERPF: effective renal plasma flow; hANP: human atrial natriuretic peptide.

Acute post-streptococcal glomerulonephritis (APSGN) is generally classified as intrinsic acute kidney injury (AKI) ^(1)^. Although it has been reported that effective renal plasma flow (ERPF) is not decreased in APSGN, filtration fraction (FF) and glomerular filtration rate (GFR) are known to decrease ^(2)^. However, in patients with APSGN, it has been reported that fractional excretion of sodium (FENa) less than or equal to 0.5 seems to be an accurate predictor for development of hypertensive episodes ^(3)^, and both low FENa and high atrial natriuretic peptide coexist ^(4)^. They unexpectedly showed coexistence of intravascular excess fluid and reduced kidney blood flow in the acute phase of APSGN. Given ERPF constitutes approximately 90% of renal plasma flow, 90% of renal arterial blood flows sequentially from the renal arteries through the segmental, interlobar, arcuate, and interlobular arteries, then to the afferent arterioles, glomeruli, efferent arterioles, and cortical capillary plexus, and finally exits through the interlobular, arcuate, interlobar veins, and renal veins ^(5)^. There are no collateral vessels in this system. APSGN is an endocapillary proliferative glomerulonephritis, and in its acute phase, ERPF is presumably decreased owing to endocapillary proliferation and narrowing of the glomerular vascular lumen. As shown in Figure 1, we hypothesize that oliguria in the acute phase of APSGN is driven by activation of the renin-angiotensin-aldosterone system (RAAS) in response to decreased ERPF. RAAS activation would function as a homeostatic response to maintain normal ERPF, leading to intravascular volume excess, which gradually restores ERPF. Thus, RAAS activation is expected to be transient; therefore, in actual clinical practice, it is difficult to detect the condition of hyperreninemia. The study that defined the acute phase over an extended duration (within a month of onset of the disease) may not accurately assess ERPF in APSGN owing to the inclusion of varying pathophysiological states ^(2)^. In typical APSGN, hypertension and edema improve with diuresis within one to two weeks ^(6)^.

This retrospective case series study aims to show the possibility that the pathogenesis of oliguria in APSGN is due to RAAS activation caused by reduced renal blood flow, similar to prerenal AKI. By appropriately staging the acute phase of APSGN and retrospectively analyzing physical and laboratory findings, we seek to elucidate the seemingly paradoxical condition of fluid overload and reduced renal blood flow during the acute phase of APSGN.

## Materials and Methods

### Ethics statement

All procedures involving human participants were in accordance with the ethical standards of the institution at which the studies were conducted (approval number in Japanese Red Cross Aichi Medical Center Nagoya Daini Hospital: 1385S), and with standards of the Declaration of Helsinki of 1964 and its later amendments or comparable ethical standards. Written informed consent was not obtained because of the use of retrospective data for clinical use. This information is available on the website (opt-out).

### Study population

We included patients with APSGN aged ≤15 years admitted to our hospital and seven affiliated hospitals from 2010 to 2019. APSGN was defined as meeting all the following criteria ^(1), (7), (8)^:

1. Anti-streptolysin O antibody ≥350 IU/mL.

2. Hypertension: systolic or diastolic blood pressure above the 95th percentile, based on the 2017 Clinical Practice Guidelines from the American Academy of Pediatrics ^(9)^.

3. Hematuria: urine occult blood ≥1+ or erythrocyte sediment ≥5 per high-power field.

4. Proteinuria: urine protein qualitative ≥1+ or urine protein-to-creatinine ratio ≥0.15 g/g creatinine.

5. C3 <70 mg/dL with recovery to >70 mg/dL within two months.

In addition, only those diagnosed with APSGN who had fluid overload―defined as a weight gain of at least 5% greater than their baseline body weight and brain natriuretic peptide (BNP) ≥100 pg/mL―were included. The reason is that we would like to include patients with moderate or severe disease who experienced intravascular excess fluid to understand the characteristics of the acute phase of APSGN. The lowest weight measured during observation was considered the baseline body weight.

The peak period was defined as three days: the day of highest weight and one day before and after. The subsequent two days were defined as the early recovery period, and the further period up to 30 days as the late recovery period (Figure 1). Patients with assessable FENa and BNP measurements in at least two disease periods were included.

### Data collection

We retrospectively analyzed the corresponding patients for age, sex, body weight, body surface area, urine occult blood, urine protein-to-creatinine ratio, urine β2-microglobulin, blood pressure, creatinine-based estimated GFR (eGFR), complement component 3, anti-streptolysin O antibody, BNP, FENa, cardiothoracic ratio (CTR), and inferior vena cava diameter (IVCd). The relationship between renal blood flow and fluid overload was examined specifically for discrepancies during the peak period because renal blood flow is generally higher during periods of high fluid overload. Primarily, FENa was used as an indicator of renal blood flow and BNP as an indicator of fluid overload. Furosemide was administered in four patients. Although FENa is affected by furosemide, data after furosemide administration during the peak phase were excluded. There was no use of furosemide during the late recovery phase.

In this case series, BNP was defined as an indicator of intravascular excess fluid, and FENa as an indicator of reduced renal blood flow. Ideally, intravascular excess fluid should be evaluated comprehensively using BNP, CTR, and IVCd. However, because only BNP was assessed over time, BNP, CTR, and IVCd were similarly increased in most acute-phase cases, and none of the patients had pre-existing heart failure, we considered it reasonable to track BNP over time. FENa is widely used to assess kidney function, particularly in AKI, although its application is not limited to this condition. A decrease in GFR alone is unlikely to cause a decrease in FENa without prerenal factors. When tubular function is intact, FENa can serve as an indicator of reduced renal blood flow. To confirm that, we reviewed the urine β2-microglobulin data. Given we did not directly measure renal plasma flow, we used FENa as a surrogate marker for renal blood flow. We used the eGFR as a surrogate for GFR. The eGFR was calculated using Uemura’s formula ^(10)^ based on serum creatinine levels, sex, and body length.

### Statistical analysis

SPSS version 26 was used for statistical analysis, and the significance level was set at 0.05. The Wilcoxon signed-rank test was used to evaluate the changes in each parameter.

## Results

**Figure 2.**
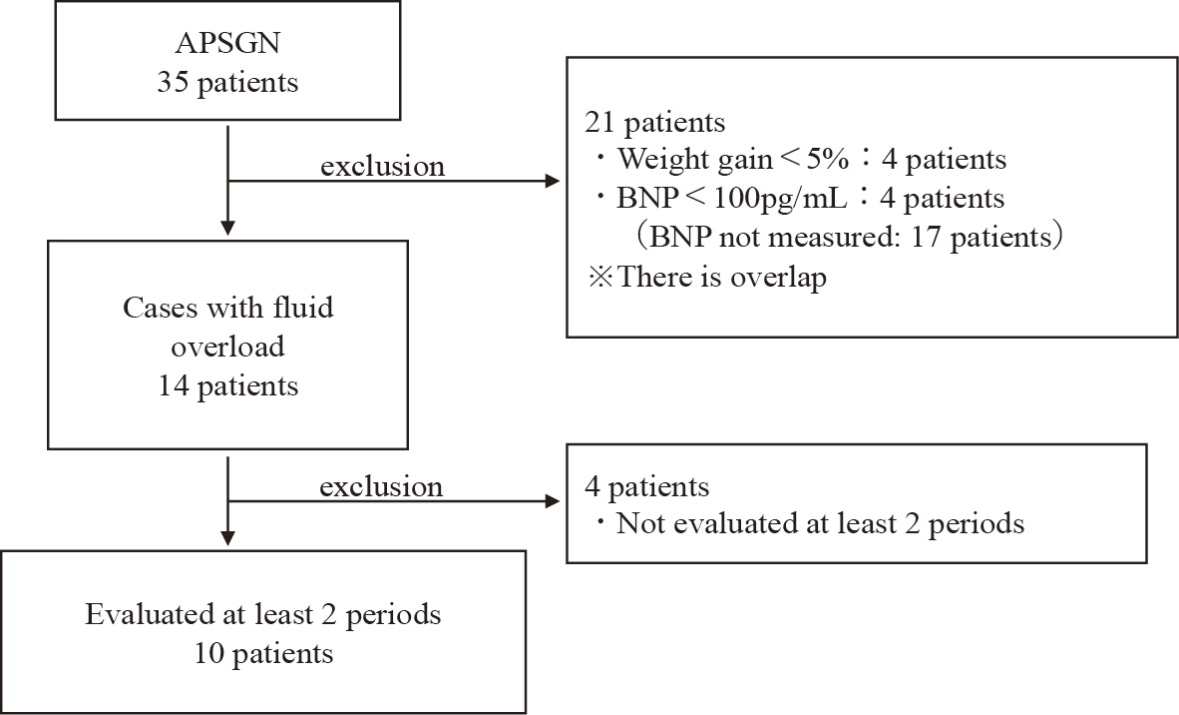
Flowchart illustrating the inclusion/exclusion of individuals in the study. APSGN: acute post-streptococcal glomerulonephritis; BNP: brain natriuretic peptide.

There were 35 hospitalized patients diagnosed with APSGN at the four hospitals. The other three facilities did not have any patients who met the above inclusion criteria. Of the 35 patients hospitalized, 14 met the definition of fluid overload. Data from 10 patients who had measurements in at least two disease periods were included in the study (Figure 2). The characteristics of these 10 patients and data at disease onset are listed in Table 1. There were five males and five females, with a median age of 7 years (interquartile range [IQR]: 5-7). The median (IQR) values of FENa, BNP, IVCd, IVCd/m^2^, and CTR during the peak period were 0.20% (0.12-0.54), 337 pg/mL (144-628), 12 mm (9.9-15.4), 17.4 mm/m^2^ (12.8-19.2), and 52% (51-54), respectively. IVCd was measured in seven patients during the peak period, and IVCd/m^2^ exceeded the reference value of 7.0 ± 2.1 mm/m^2^ for normal children in all cases ^(10)^. CTR was measured in nine patients, and seven showed above the normal range for their age ^(11)^.

**Table 1.** Characteristics and Data at Disease Onset.

Patients	Age	Sex	UOB	UPCR	UB_2_MG	BP	Cr-eGFR	C3	ASO	FENa	FEUN	BNP	IVCd	IVCd/m^2^	CTR(%)	CTR(%)
	(yrs)			(mg/g)	μg/L	(mmHg)	(ml/min/1.73m^2^)	(mg/dL)	(IU/mL)	(%)	(%)	(pg/mL)	(mm)	(mm/m^2^)		Normal range by age
1	7	M	2+	1.30	58	147/98	79.7	27	696	0.12	10.7	231	NA	NA	52	43-49
2	5	F	3+	3.90	224	112/76	88.6	10	775	0.2	26.3	150	14.5	20.1	51	40-52
3	7	M	3+	0.49	10	140/90	65.8	18	1213	NA	19.2	401	18	18.9	53	43-49
4	5	F	3+	0.67	187	132/92	97.7	11	1109	0.19	21	606	13	18.6	57	40-52
5	7	M	4+	6.10	54	164/104	41.6	7	1090	0.1	14.3	944	10	12.8	55	43-49
6	7	F	4+	1.78	64	152/98	71.0	14	1884	0.58	17.7	315	NA	NA	54	44-50
7	8	M	1+	0.35	243	150/99	102.4	17	593	NA	24.1	170	NA	NA	50	42-49
8	6	F	4+	0.77	30	150/92	70.6	20	556	0.32	13.7	694	9.6	12.8	NA	40-50
9	4	M	3+	5.69	162	131/95	48.0	25	509	0.54	23.2	358	11	16.2	52	40-52
10	10	F	3+	0.60	40	142/93	83.6	13	381	NA	14.4	126	NA	NA	51	43-49
Median	7			1.04	61		75.4	16	736	0.2	18.4	337	12	17.4	52	
IQR	5-7			0.57-4.35	38-196		61.4-90.9	11-21	544-1135	0.12-0.54	14.2-23.4	144-628	9.9-15.4	12.8-19.2	51-54	

ASO: anti-streptolysin O antibody; BNP: brain natriuretic peptide; BP: blood pressure; C3: complement component 3; Cr-eGFR: creatinine-based estimated glomerular filtration rate; CTR: cardiothoracic ratio; FENa: fractional excretion of sodium; FEUN: fractional excretion of urea nitrogen; IQR: interquartile range; IVCd: inferior vena cava diameter; NA: not available; UB_2_MG: urine β2-microglobulin; UOB: urine occult blood; UPCR: urine protein-to-creatinine ratio.

Table 2 lists FENa and BNP in three periods of peak, early recovery, and late recovery; in the table, we show the renal blood flow as FENa and the intravascular excess fluid as BNP. The median FENa values in the peak and late recovery phases were 0.198 and 0.533, respectively (p = 0.047). The median values of BNP in the peak and late recovery phases were 357.6 and 22.9, respectively (0.012). As the phase progresses, the coexistence of the seemingly contradictory increased fluid volume and decreased renal blood flow during the peak phase improved over a short period, as presented in Table 2.

**Table 2.**
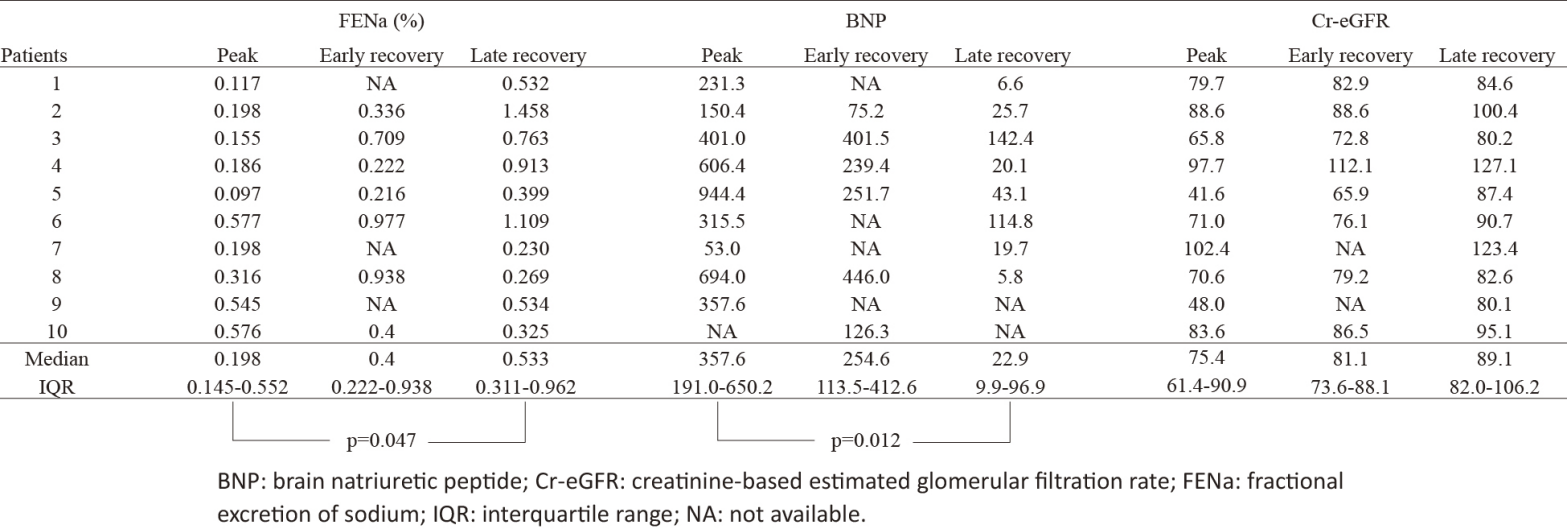
FENa, BNP, and Cr-eGFR at Three Periods.

## Discussion

In this retrospective study, we implied that during the peak period of APSGN, low values of FENa indicated decreased renal blood flow, and high values of BNP, IVCd/m^2^, and CTR showed increased circulating blood volume, and in addition, we show that the seemingly contradictory disease states of fluid overload and reduced renal blood flow improve during the clinical recovery phase, by observing changes in FENa and BNP with disease stage. Incidentally, urinary β2-microglobulin as a method for evaluating tubular function, which was investigated to increase the reliability of FENa, was within the normal range in all cases (Table 1).

At the peak of APSGN, FENa was low, but blood pressure, BNP, IVCd, and CTR were high. Blood pressure was stage 2 hypertension except in case 2, and considering the BNP, IVCd, and CTR data, this hypertension was considered volume-dependent hypertension. However, if this was the initial event, it would be inconsistent with the reduced renal blood flow expected given the low FENa. As shown in Figure 1, it is more plausible that a decrease in renal blood flow occurred first, followed by a temporary increase in RAAS and intravascular extravasation as a homeostatic event.

FENa decreases when the RAAS is activated ^(12)^. BNP, in contrast, has natriuretic and RAAS inhibitory effects ^(13)^. In general, ERPF and FENa would increase if fluid overload occurred, as indicated by elevated BNP and increased IVCd and CTR. However, in the present findings, FENa remained low despite sufficient fluid overload at the peak. Furthermore, the low FENa levels and high BNP levels improved significantly between the peak and late recovery periods (Table 2). This suggests our hypothesis presented in Figure 1, namely, (1) during the peak phase of APSGN, ERPF decreases due to endothelial cell swelling, leading to transient activation of the RAAS; (2) sodium and water are reabsorbed from the renal tubules, causing fluid overload (elevated BNP, IVCd, CTR); (3) the renal tubules are flooded with sodium and water, causing hypertension (increased BNP, IVCd, CTR); (4) APSGN is a self-limited acute disease that resolves as the endothelial cell swelling subsides; and (5) a temporary increase in ERPF occurs during recovery, leading to a diuretic phase. The pathology of oliguria, which is an initial symptom of the disease, is similar to that of prerenal AKI, and although increasing systemic vascular volume is a goal in general prerenal AKI, the purpose of APSGN is to prevent acute tubular necrosis only targeting the kidneys. Once fluid overload is created by these responses and renal blood flow is maintained, RAAS will likely normalize rapidly. In early recovery, as endothelial cell swelling decreases and ERPF improves, the RAAS normalizes and sodium and water reabsorption from the renal tubules decreases. This explains the observed increase in FENa during this period, whereas BNP levels decreased. The wide range of BNP values observed during the late recovery phase was likely due to its longer duration than other phases and variations in the degree of recovery among patients.

A medical textbook notes that "renal blood flow is usually normal" in APSGN, but it does not provide citations or evidence to support this claim ^(14)^. Herthelius et al. ^(2)^ reported that although ERPF is not reduced, both FF and GFR are decreased. A criticism of that study is its definition of the acute phase as ≤30 days post-onset of the disease (mean, 16 days). Given urine output typically increases within 5-10 days in APSGN, with accompanying improvements in hypertension and edema ^(14)^, defining the first 30 days as the acute phase causes patients to be assessed at various stages, from truly acute to convalescent. Therefore, in this study, we shortened the peak period and the early recovery period to assess changes in blood flow during the acute phase. Our results suggest that volume overload and tubular reabsorption profiles change within just a few days of transitioning from the peak period to the early recovery period. The true acute phase should not be measured in weeks but in days. To the best of our knowledge, this study is likely the first to examine renal blood flow during the true acute phase of APSGN.

Because most cases of APSGN resolve spontaneously, kidney biopsies should be avoided, with only supportive therapies such as fluid and salt restriction and antihypertensive medications being used. However, if other forms of nephritis are suggested, a kidney biopsy may be warranted, and in some cases, immunosuppressive therapy might be necessary. A correct understanding of the pathogenesis of acute oliguria in APSGN will aid in the proper and safe management of these patients. Moreover, this understanding is crucial for the appropriate management of diseases that cause endocapillary proliferation, not only APSGN. It would be meaningful to show that the acute phase of APSGN involves the seemingly contradictory pathological condition of fluid overload and reduced renal blood flow.

This study has several limitations. First, it is a case series with a small number of patients; furthermore, as seen in Table 2, missing data are present, making drawing robust conclusions difficult. However, FENa values in the peak and late recovery phases have no missing data, and BNP values collected during the same phases have only two missing values; therefore, we believe that FENa and BNP values during these phases are fairly reliable. In addition, the study's design, focusing only on patients with evidence of fluid overload and separating the phase into shorter periods, further limited the number of patients included, and there were also missing values because this was a retrospective study. We acknowledge the exploratory nature of this study but believe that the insights gained may be valuable for clinical practice and encourage further confirmatory research. Second, as a retrospective study, RAAS, which reflects ERPF, was not directly measured; instead, FENa was used as a proxy for ERPF. Prospective studies incorporating a large number of patients and directly measuring parameters indicative of fluid overload and RAAS would provide more robust evidence.

### Conclusions

In general, fluid overload increases renal blood flow, leading to increases in BNP and FENa. However, as shown in this study, FENa was low during the peak phase of APSGN despite high BNP, IVCd, and CTR, whereas FENa tended to increase during the recovery phase even though BNP decreased. This suggests that the acute phase of APSGN involves a paradoxical pathophysiology of fluid overload and reduced renal blood flow, whereby renal blood flow is concomitantly reduced owing to endothelial cell proliferation despite fluid overload, and renal blood flow improves during the recovery phase as endocapillary proliferation decreases.

## Article Information

### Conflicts of Interest

None

### Author Contributions

The research started with the idea of Osamu Uemura and Hisakazu Majima. All authors contributed to the conception and design of the study. Data collection was performed by Hisakazu Majima, Naoya Fujita, Katsumi Ushijima, Masamichi Miyoshi, Takuji Yamada, Masaki Yamamota, and Eiji Matsukuma. Material preparation and analysis were performed by Hisakazu Majima and Osamu Uemura. The first draft of the manuscript was written by Hisakazu Majima and Osamu Uemura, and all authors commented on previous versions of the manuscript. All authors read and approved the final manuscript.

### Approval by Institutional Review Board (IRB)

The study was approved by the ethics committee of Japanese Red Cross Aichi Medical Center Nagoya Daini Hospital (approval number: 1385S).

### Informed Consent

Written informed consent was not obtained because of the use of retrospective data for clinical use. This information is available on the website (opt-out).

## References

[1] Goldstein SL, Zappitelli M. Evaluation and management of acute kidney injury in children. In: Avner ED, Harmon WE, Niaudet P, Yoshikawa N, Emma F, Goldstein SL, editors. Pediatric nephrology. 7th ed. Vol. 3. Berlin, Germany: Springer Berlin Heidelberg; 2016. p. 2139-67.

[2] Herthelius M, Berg U. Renal function during and after childhood acute poststreptococcal glomerulonephritis. Pediatr Nephrol. 1999;13(9):907-11. 10603146 10.1007/s004670050725

[3] Mota-Hernandez F, Feiman R, Gordillo-Paniagua G. Predictive value of fractional excretion of filtered sodium for hypertension in acute post-streptococcal glomerulonephritis. J Pediatr. 1984;104(4):560-3. 6707818 10.1016/s0022-3476(84)80547-8

[4] Ozdemir S, Saatçi U, Besbas N, et al. Plasma atrial natriuretic peptide and endothelin levels in acute poststreptococcal glomerulonephritis. Pediatr Nephrol. 1992;6(6):519-22. 1482636 10.1007/BF00866489

[5] Bonsib S. Renal anatomy and histology. In: Jannette C, Silva F, Olson J, D’Agati V, editors. Heptinstall’s pathology of the kidney. 7th ed. Vol. 1. Philadelphia (PA): Lippincott Williams & Wilkins; 2016. p. 1-66.

[6] Falk R, Jennette J, Nachman P. Primary glomerular disease. In: Brenner B, editor. The kidney. 6th ed. Vol. 2. Philadelphia (PA): W B Saunders Co Ltd; 2000. p. 1263-349.

[7] Yoshizawa N, Yamakami K, Fujino M, et al. Nephritis-associated plasmin receptor and acute poststreptococcal glomerulonephritis: characterization of the antigen and associated immune response. J Am Soc Nephrol. 2004;15(7):1785-93. 15213266 10.1097/01.asn.0000130624.94920.6b

[8] VanDeVoorde RG 3rd. Acute poststreptococcal glomerulonephritis: the most common acute glomerulonephritis. Pediatr Rev. 2015;36(1):3-13. 25554106 10.1542/pir.36-1-3

[9] Flynn JT, Kaelber DC, Baker-Smith CM, et al. Clinical practice guideline for screening and management of high blood pressure in children and adolescents. Pediatrics. 2017;140(3):e20171904. 28827377 10.1542/peds.2017-1904

[10] Uemura O, Nagai T, Ishikura K, et al. Creatinine-based equation to estimate the glomerular filtration rate in Japanese children and adolescents with chronic kidney disease. Clin Exp Nephrol. 2014;18(4):626-33. 24013764 10.1007/s10157-013-0856-y

[11] Sönmez F, Mir S, Ozyürek AR, et al. The adjustment of post-dialysis dry weight based on non-invasive measurements in children. Nephrol Dial Transplant. 1996;11(8):1564-7. 8856212

[12] Keats T. The cardiovascular system. In: Keats T, Lusted L, editors. Atras of Roentgenographic measurement. 5th ed. Chicago (IL): Mosby; 1985. p. 262-314.

[13] Castle-Kirszbaum M, Kyi M, Wright C, et al. Hyponatraemia and hypernatraemia: disorders of water Balance in Neurosurgery. Neurosurg Rev. 2021;44(5):2433-58. 33389341 10.1007/s10143-020-01450-9

[14] Brunner-La Rocca HP, Kaye DM, Woods RL, et al. Effects of intravenous brain natriuretic peptide on regional sympathetic activity in patients with chronic heart failure as compared with healthy control subjects. J Am Coll Cardiol. 2001;37(5):1221-7. 11300426 10.1016/s0735-1097(01)01172-x

